# Are healthcare costs from obesity associated with body mass index, comorbidity or depression? Cohort study using electronic health records

**DOI:** 10.1111/cob.12144

**Published:** 2016-04-21

**Authors:** C. Rudisill, J. Charlton, H. P. Booth, M. C. Gulliford

**Affiliations:** ^1^ Department of Social PolicyLondon School of Economics and Political ScienceLondonUK; ^2^ Department of Primary Care and Public Health SciencesKing's College LondonLondonUK; ^3^ NIHR Biomedical Research Centre at Guy's and St Thomas’ NHS Foundation TrustLondonUK

**Keywords:** Comorbidity, depression, healthcare costs, obesity

## Abstract

The objective of this study was to evaluate the association between body mass index (BMI) and healthcare costs in relation to obesity‐related comorbidity and depression. A population‐based cohort study was undertaken in the UK Clinical Practice Research Datalink (CPRD). A stratified random sample was taken of participants registered with general practices in England in 2008 and 2013. Person time was classified by BMI category and morbidity status using first diagnosis of diabetes (T2DM), coronary heart disease (CHD), stroke or malignant neoplasms. Participants were classified annually as depressed or not depressed. Costs of healthcare utilization were calculated from primary care records with linked hospital episode statistics. A two‐part model estimated predicted mean annual costs by age, gender and morbidity status. Linear regression was used to estimate the effects of BMI category, comorbidity and depression on healthcare costs. The analysis included 873 809 person‐years (62% female) from 250 046 participants. Annual healthcare costs increased with BMI, to a mean of £456 (95% CI 344–568) higher for BMI ≥40 kg m^−2^ than for normal weight based on a general linear model. After adjusting for BMI, the additional cost of comorbidity was £1366 (£1269–£1463) and depression £1044 (£973–£1115). There was evidence of interaction so that as the BMI category increased, additional costs of comorbidity (£199, £74–£325) or depression (£116, £16–£216) were greater. High healthcare costs in obesity may be driven by the presence of comorbidity and depression. Prioritizing primary prevention of cardiovascular disease and diabetes in the obese population may contribute to reducing obesity‐related healthcare costs.

**What is already known about this subject?**
- Obesity is associated with higher healthcare costs.
- It is less clear whether obesity, or obesity‐associated comorbidities and depression, are the main driver of healthcare costs.
- Few studies have been based on nationally representative data sources.

**What this study adds?**
- This study reports data for a large English cohort, with 873 809 person‐years of follow‐up.
- Healthcare costs are greater as the BMI category increases, but comorbidity and depression are the greatest drivers of healthcare costs in obesity.
- Prioritizing primary prevention of cardiovascular disease and diabetes, as well as improving depression management in obesity, may contribute to reducing obesity‐related healthcare costs.

## Introduction

Obesity is a growing global health concern, accounting for substantial national healthcare expenditures, with healthcare costs predicted to be higher by around a third in obese people compared to those of normal weight 1. The association between obesity and healthcare costs is well‐documented in international literature. Investigators have largely used attributable fraction methodology 2, 3 and, more recently, instrumental variable approaches.^4^, ^5^. These studies have estimated the proportion of healthcare spending on obesity to be around 5%, with results of up to 20% identified in the United States 4, 6. Despite recent efforts to quantify direct costs associated with obesity, the mediators underlying this relationship are poorly understood. Given the continued rise in obesity prevalence and persistence of the condition in individuals 7, the drivers of obesity‐related costs need to be analysed.

The aim of this paper is to investigate the association between body mass index (BMI) category and healthcare costs, focusing on the issue of whether BMI category, obesity‐related comorbidity and/or depression most strongly determines costs related to obesity. Answering this question may enable better informed efforts to increase the effectiveness and efficiency of the management of obese individuals. This analysis advances the literature on obesity‐related healthcare costs in three important ways. First, it utilizes data from a large and nationally representative database. Previous studies have relied on smaller 4, regional sources 5 of healthcare data to estimate costs. Secondly, we have controlled for depression separately from other obesity‐related comorbidities. Depression may be associated with higher costs either in obesity or in comorbidity. We know that the obese have higher odds of depression 8. This study aimed to estimate the effect of weight on costs separate from that of depression. Third, this study offers estimates for patient‐level costs of obesity using individual‐level patient data.

## Methods

We undertook a population‐based cohort study using the UK Clinical Practice Research Datalink (CPRD) (https://www.cprd.com). The CPRD collects primary care electronic health records for 7% of the UK population and is considered representative of the UK population in terms of patient demographic characteristics and the size and composition of the general practices of reporting data 9. This research was part of a larger project to evaluate the cost‐effectiveness of bariatric surgery in adults, and participants aged 20 or older were included.

### Study design and participant selection

A cohort of participants was drawn from the CPRD. Participants were eligible if they were registered with a CPRD general practice between 1 January 2008 and 31 December 2013. We evaluated eligible participants’ BMI records using the categories 18.5–24.9; 25.0–29.9; 30.0–34.9; 35.0–39.9; 40.0–44.9 and 45.0 kg m^−2^ and above. We took a random sample of up to 50 000 participants from each BMI category. This gave a sample of 259 007 participants; 1819 participants who received bariatric surgery were excluded, leaving 257 188 participants for further analysis. For the present analyses, we further restricted the participants to those registered with CPRD practices in England that participated in the data linkage scheme, providing associated data for Hospital Episode Statistics (HES). This left 250 046 participants for the analysis.

### Statistical data analysis

Electronic health records were analysed from the latest of the date the practice joined CPRD or the patient's first registration to the earliest of the end of patient registration or date of death. Person time for men and women was allocated to a BMI category according to the most recent BMI value, combining BMI records >40 Kg m^−2^ into a single group to represent morbid obesity. Comorbidity status was evaluated using medical diagnoses coded into electronic health records during general practice consultations. Person time was classified according to morbidity status using the first diagnosis of type 2 diabetes (T2DM), coronary heart disease (CHD), stroke or cancer. Depression was re‐evaluated in each year of follow‐up, and patients were considered to have depression if they were diagnosed with depression during the year or if they were prescribed antidepressants in year and were ever diagnosed with depression. Morbidities, including T2DM 10, CHD 11, stroke 12 and depression 13 were evaluated using medical codes reported previously, while cancer diagnoses were evaluated using codes for malignant neoplasms.

Health care utilization was estimated from participants’ electronic health records with linked hospital episode statistics (HES) data. Primary and secondary care utilization was evaluated, including primary care consultations at the practice, by telephone, at home, emergency and out‐of‐hours. Secondary care utilization included admissions to hospital, out‐patient, day case and emergency visits. All drug prescriptions issued by the practice were evaluated. Utilization rates were calculated using person time at risk. Age‐standardized rates were estimated using direct standardization and the European Standard Population (ESP) for reference. The costs of healthcare utilization were evaluated for participants by morbidity and depression status within BMI categories for the period 2008–2013. Initially, the mean cost per person per year was calculated for each combination of BMI category and comorbidity status. The analysis was undertaken from a health service provider perspective (UK National Health Service) and did not consider indirect costs such as loss of productivity or patient and carer time.

Unit costs were applied to the categories of healthcare utilization to estimate healthcare costs. The unit costs were all taken from reference sources based on UK£ 2013 price estimates. The Personal Social Service Research Unit (PSSRU) publication ‘Unit Costs of Health and Social Care (2013) was used as the reference for all healthcare costs 14. The same unit costs were applied across different levels of age group, gender, BMI category and morbidity status. Primary care general practitioner (GP) consultation, emergency or out‐of‐hours, home visit and telephone consultations were priced at £45, £45, £114 and £27, respectively. Unit costs of secondary care in‐patient, out‐patient, day‐case and emergency visits were priced at £1400, £135, £697 and £135, respectively. To assess prescription costs, Gemscript drug codes for prescriptions in the electronic health records were linked with prescription costs from a dictionary compiled by RESIP UK (Chertsey, Surrey, UK).

A two‐part model 15, 16 was used to analyse healthcare costs. In the first stage, a probit model was employed to estimate the probability of healthcare utilization being non‐zero. In the second stage, a generalized linear model (GLM), with a log link and gamma errors, was used to evaluate the distribution of costs in participants who utilized health care. This predicted mean costs of care, as the product of the probability of utilizing care and mean costs of care, for men and women in different BMI and morbidity categories for each year of age. In the final stage of analysis, a linear regression model was employed to estimate the effects of the BMI category, comorbidity and depression on predicted healthcare costs, controlling for patient gender and age. Interaction terms for comorbidity and depression and comorbidity and BMI category were included. In order to make the data sufficiently concise for presentation, the comorbidities of T2DM, CHD, stroke and cancer were combined into a single category of ‘comorbidity’ present or absent for the linear regression analysis. All statistical analyses were performed using Stata 13 for Windows (Stata Corporation, College Station, TX, USA).

### Study approval

The research represents part of a study approved by the Independent Scientific Advisory Committee (ISAC) of the Medicines and Healthcare products Regulatory Agency (MHRA) (ISAC Protocol No. 13–089).

## Results

The cohort person‐years are presented in Table 1. Women (62%) contributed more person time than men (38%), but person time was distributed evenly among age groups and BMI categories. Diabetes and depression were each prevalent in 16% of person‐years.

**Table 1:** Distribution of person years by body mass index category, comorbidity and depression

Variable	Category	Person‐years	Percent of total
Total		873 809	(100)
Gender	Male	335 610	(38)
	Female	538 199	(62)
Age group	20–34	118 364	(14)
	35–44	151 810	(17)
	45–54	170 562	(20)
	55–64	170 362	(19)
	65–74	143 723	(16)
	75–84	90 972	(10)
	85+	28 017	(3)
BMI category	18.5–24.9	130 806	(15)
	25.0–29.9	171 622	(20)
	30.0–34.9	181 283	(21)
	35.0–39.9	173 470	(20)
	40.0+	216 629	(25)
Comorbidity	None	573 998	(66)
	T2DM	144 089	(16)
	CHD	76 144	(9)
	Stroke	19 978	(2)
	Cancer	59 600	(7)
Depression	Not depressed	731 526	(84)
	Depressed	142 282	(16)

The two‐part model used to estimate healthcare costs is presented in Table 2. Coefficients associated with making any use of health care (probit model) increased with BMI category and were also elevated in the presence of comorbidity. Coefficients associated with healthcare costs among those utilizing care also increased with BMI category and were elevated in comorbidity and depression. Interactions terms for the effect of BMI category by comorbidity and comorbidity by depression on predicted healthcare costs were significant in both the probit and GLM models.

**Table 2:** Two‐part regression model for healthcare costs (UK£2013)

Predictor		Probit model		GLM model	
		Coefficient (95% confidence interval)	*P*‐Value	Coefficient (95% confidence interval)	*P*‐Value
Age	Per year	−0.019 (−0.022 to −0.17)	<0.001	−0.017 (−0.020 to −0.015)	
Age squared		0.00034 (0.00031–0.00037)	<0.001	0.00027 (0.00025–0.00030)	<0.001
Gender	Female	0.033 (0.031–0.034)		0.02 (−0.00 to 0.03)	0.094
BMI category	18.5–24.9	Ref.		Ref.	
	25.0–29.9	0.08 (0.06–0.10)	<0.001	0.01 (−0.02 to 0.03)	0.660
	30.0–34.9	0.18 (0.16–0.20)	<0.001	0.11 (0.08–0.14)	<0.001
	35.0–39.9	0.21 (0.19–0.23)	<0.001	0.18 (0.15–0.21)	<0.001
	40.0+	0.25 (0.23–0.27)	<0.001	0.29 (0.26–0.32)	<0.001
Depression	Present	1.68 (1.56–1.80)	<0.001	0.61 (0.54–0.67)	<0.001
Comorbidity	None	Ref.		Ref.	
	T2DM	0.64 (0.53–0.75)	<0.001	0.84 (0.70–0.98)	<0.001
	Coronary heart disease	0.54 (0.44–0.65)	<0.001	0.79 (0.69–0.89)	<0.001
	Stroke	0.35 (0.21–0.49)	<0.001	1.01 (0.81–1.21)	<0.001
	Cancer	0.35 (0.28–0.43)	<0.001	0.84 (0.78–0.89)	<0.001
BMI*depression		χ^2^ = 11.9, df = 4	0.018	χ^2^ = 1.6, df = 4	0.810
BMI*comorbidity		χ^2^ = 33.6, df = 16	0.006	χ^2^ = 54.5, df = 16	<0.001
Comorbidity*depression		χ^2^ = 112.4, df = 4	<0.001	χ^2^ = 130.1, df = 4	<0.001

BMI, body mass index; CHD, coronary heart disease; Ref., reference category.

The predicted annual healthcare costs in UK£ by BMI category, gender, comorbidity and depression status are presented in Table 3. Overall, there is a largely positive linear relationship between healthcare costs and BMI category. Depression was consistently associated with greater healthcare costs in all states. Comorbidity was associated with considerably greater costs at any level of BMI. However, there are some notable exceptions. Normal weight was noted to be associated with higher predicted healthcare costs than overweight. This result is most evident in participants with T2DM. In men with depression and T2DM, normal weight participants had the highest estimated cost of all BMI categories, at £3940 compared to £3796 in the morbidly obese category. Similarly, in men with stroke and depression the annual estimated cost was highest in the normal weight category, at £4442 compared to £4385 in the morbidly obese category. Strokes appear to be related to the highest overall costs with the exception of men who are not depressed and who have a BMI ≥ 25 who have been diagnosed with cancer and women who are not depressed and who are obese and have been diagnosed with cancer.

**Table 3:** Predicted annual healthcare costs (UK£ 2013) by BMI category, gender, depression and comorbidity

Gender	Depression		BMI category (kg m^−2^)				
			18.5–24.9	25–29.9	30–34.9	35–39.9	40+
Men	Not depressed	At risk	997	1016	1134	1206	1376
		DM	2471	2242	2267	2356	2559
		CHD	2341	2121	2469	2602	2871
		Stroke	2864	2551	2882	2962	3074
		Cancer	2406	2566	2936	3059	3280
	Depressed	At risk	1985	1981	2128	2282	2506
		DM	3940	3527	3530	3621	3796
		CHD	3645	3204	3719	3876	4227
		Stroke	4422	3876	4339	4343	4385
		Cancer	3458	3690	4097	4227	4274
Women	Not depressed	At risk	1057	1072	1194	1289	1439
		DM	2548	2306	2332	2421	2659
		CHD	2421	2177	2537	2667	3061
		Stroke	2983	2676	3020	3054	3229
		Cancer	2505	2662	3040	3162	3419
	Depressed	At risk	2040	2029	2225	2441	2602
		DM	4007	3588	3617	3786	4055
		CHD	3741	3276	3810	4001	4513
		Stroke	4492	3969	4407	4528	4750
		Cancer	3577	3759	4207	4435	4527

Figures are mean values across ages 20–100 years.

BMI, body mass index; CHD, coronary heart disease; T2DM, diabetes mellitus.

Table 4 shows mean differences in healthcare costs estimated from a linear regression model. The presence of a comorbidity was identified as the single largest predictor of healthcare costs, with a £1366 (95% CI £1296–£1463) mean increase in annual patient costs if morbidity was present. The second greatest predictor was depression, at £1044 (£973–£1115) per patient per year. The BMI category was positively associated with healthcare costs but at a much smaller magnitude, with a £456 increase in morbidly obese participants compared to normal weight. Being overweight did not increase average healthcare costs. The female gender was related to higher costs but of a lower magnitude (£113 in females), while the expected increase in costs with age was observed. Finally, there was evidence of a multiplicative effect on costs, with the presence of depression or high BMI alongside a comorbidity. Having depression alongside a comorbidity was associated with an additional cost of £243 (£164–£322) per year, while being obese, severely obese or morbidly obese increased costs by a further £168, £152 or £199, respectively. Depression had a greater additive effect on cost in conjunction with a comorbidity than BMI. The additive cost impact of a comorbidity was elevated for those at normal weight.

**Table 4:** Estimated effects of gender, age, depression BMI group and comorbidity on annual healthcare costs (UK£ 2013)

Variable	Category	Adjusted mean difference (£) (95% confidence interval)	*P*‐Value
Gender	Male	Ref.	
	Female	113 (81–144)	<0.001
Age	per year	51 (50–52)	<0.001
Depression	Not depressed	Ref.	
	Depressed	1044 (973–1115)	<0.001
BMI category		Ref.	
	25–29	5 (−117 to 107)	0.934
	30–34	146 (34–258)	0.011
	35–39	280 (168–392)	<0.001
	40+	456 (344–568)	<0.001
Comorbidity*	Absent	Ref.	
	Present	1366 (1269–1463)	<0.001
Comorbidity × depression†		243 (164–322)	<0.001
Comorbidity × BMI category‡	18.5–24	232 (106–35)	<0.001
	25–29	Ref.	
	30–34	168 (43–293)	0.009
	35–39	152 (17–277)	0.017
	40+	199 (74–325)	0.002
BMI category × depression	18.5–24	121 (20–221)	0.018
	25–29	Ref.	
	30–34	76 (−24 to 176)	0.137
	35–39	125 (25–225)	0.014
	40+	116 (16–216)	0.024

* Includes T2DM, coronary heart disease, stroke or cancer.

† Additional cost associated with co‐occurrence of comorbidity and depression.

‡ Additional cost associated with co‐occurrence of comorbidity and stated BMI category.

## Discussion

This study investigated healthcare costs in relation to BMI and may be the first study to isolate depression as a driver of costs separate from other common obesity‐related morbidities. Previous studies investigating healthcare costs relating to obesity using patient cohorts have attempted to estimate the marginal effect of obesity on costs 4, 5. Understanding the causal association between BMI and healthcare utilization is important, but in the context of treating patients in a clinical environment, there is little benefit in divorcing obesity from related morbidities and socioeconomic factors.

There is a wide variation in cost estimates for obesity based on population sample studies, ranging from an additional €160 per year for severe obesity, compared to normal weight 5, to $2741 for any obesity compared to normal weight 4. Our findings estimated an increase of £146 per year for simple obesity, in comparison with normal weight, and £456 for morbid obesity. These estimates were for the influence of BMI separate from the considerations of obesity‐related morbidities and depression.

We found that having a comorbidity was the greatest predictor of increased healthcare costs at £1366 per year followed by clinical depression at £1044 per year. Of the four obesity‐related morbidities we assessed, stroke was found to be associated with the highest absolute costs, which is likely to result from acute secondary care required after a stroke. This finding is supported by a previous paper that identified cardiovascular diseases and cardiovascular agents as the biggest drivers of healthcare claims costs per unit of BMI 17.

We found that depression was the second greatest driver of costs, and its presence increased costs in all conditions, consistent with a previous investigation conducted into depression and healthcare utilization 13. The impact of depression alongside obesity on healthcare costs has been reported in a previous study based on secondary care 18. Overall, having an elevated BMI category in addition to a comorbidity appeared to increase costs, although having depression was an even greater contributor to high healthcare costs alongside a comorbidity. Being overweight was not associated with higher healthcare costs than normal weight. The lack of statistically significant difference in costs between normal and overweight BMI categories has also been found in other studies 6, 19.

In T2DM, body weight did not appear to be associated with healthcare costs. This phenomenon has been reported elsewhere, and the possibility of greater morbidity in diabetics who are of normal weight was suggested as an explanatory factor 20, 21. Our analyses did not standardize for the duration of T2DM or the extent of diabetes‐related complications.

This study has a number of strengths that make it a valuable addition to the literature on health care‐related costs of obesity. Firstly, it is based on a large, longitudinal, nationally representative dataset and uses costs associated directly with provision of care rather than based on insurance claims, which are an indirect measure of morbidity and may be less accurate. It is also one of few papers to use clinically measured height and weight values in the calculation of BMI rather than self‐reported values that are prone to reporting bias 22. This is also the only paper we are aware of to use UK sample data to estimate the healthcare costs associated with obesity. One possible limitation of the study is selection bias from inclusion of participants with a BMI recorded during a clinical consultation, who may be more frequent users of healthcare services and therefore less healthy. Participants’ morbidity status was classified using the first diagnosis they received, and we have not accounted for additional diagnoses. The costs associated with the conditions we have highlighted may represent costs from multiple morbidities, which are more frequent in obese patients and we know were present in 18% of the observations 23.

The economics literature has moved towards using an instrumental variables approach to examine the causal effect of obesity on medical costs by using the weight of a biological relative as an instrument 4, 5. The argument for this method is that the instrument predicts the participants’ weight but not their morbidity status, meaning that the effect of weight on costs can be isolated. Such papers have found higher healthcare costs for obesity than non‐instrumented methods, so it is possible our models underestimate the magnitude of the relationship.

The findings of this analysis emphasize that healthcare costs for obesity are largely driven by a few key obesity‐related morbidities and not by high BMI alone. Further research is now needed to elucidate the costly elements of these patients’ care and to develop clinical pathways to manage them more efficiently. Persistently high obesity rates and evidence from the literature 24 suggest that preventive and reactive treatment strategies for obesity are currently of limited effectiveness. Until greater success is observed in lowering obesity levels, the clinical focus should be on prevention of secondary conditions as a way of improving the health status of obese patients and lowering resulting costs. Increasing the uptake of successful T2DM prevention programmes 25 may prove particularly beneficial given the high costs observed across BMI categories in this analysis. Similarly, successful interventions to focus on the mental health of the obese might also stem resultant health and non‐healthcare costs related to depression.

## Conflicts of Interest Statement

All authors have completed the ICMJE uniform disclosure form at www.icmje.org/coi_disclosure.pdf and declare no support from any organization for the submitted work; no financial relationships with any organizations that might have an interest in the submitted work in the previous 3 years; and no other relationships or activities that could appear to have influenced the submitted work.

## Author contributions

MG and ACR designed the study. JC and MG conducted the analysis. ACR and MG interpreted the results. MG, ACR and HB drafted the paper. All authors commented on and approved the manuscript.
